# Estimating the effect size of surgery to improve walking in children with cerebral palsy from retrospective observational clinical data

**DOI:** 10.1038/s41598-018-33962-2

**Published:** 2018-11-05

**Authors:** Apoorva Rajagopal, Łukasz Kidziński, Alec S. McGlaughlin, Jennifer L. Hicks, Scott L. Delp, Michael H. Schwartz

**Affiliations:** 1 Department of Mechanical Engineering, Stanford University, Stanford, CA USA; 2 Department of Bioengineering, Stanford University, Stanford, CA USA; 3 Center for Gait and Motion Analysis, Gillette Children’s Specialty Healthcare, St. Paul, MN USA; 4 Department of Orthopedic Surgery, University of Minnesota, Minneapolis, MN USA

## Abstract

Single-event multilevel surgery (SEMLS) is a standard treatment approach aimed at improving gait for patients with cerebral palsy, but the effect of this approach compared to natural progression without surgical intervention is unclear. In this study, we used retrospective patient history, physical exam, and three-dimensional gait analysis data from 2,333 limbs to build regression models estimating the effect of SEMLS on gait, while controlling for expected natural progression. Post-hoc classifications using the regression model results identified which limbs would exhibit gait within two standard deviations of typical gait at the follow-up visit with or without a SEMLS with 73% and 77% accuracy, respectively. Using these models, we found that, while surgery was expected to have a positive effect on 93% of limbs compared to natural progression, in only 37% of limbs was this expected effect a clinically meaningful improvement. We identified 26% of the non-surgically treated limbs that may have shown a clinically meaningful improvement in gait had they received surgery. Our models suggest that pre-operative physical therapy focused on improving biomechanical characteristics, such as walking speed and strength, may improve likelihood of positive surgical outcomes. These models are shared with the community to use as an evaluation tool when considering whether or not a patient should undergo a SEMLS.

## Introduction

Cerebral palsy is a motor disorder characterized by abnormal neurological and musculoskeletal development^1^. Patients exhibit high variability in both severity and presentation of the disorder^2–4^, which makes it difficult to identify the optimal treatment to improve gait. As a result, treatment outcomes are variable^5^. Gait analysis data have been used for decades to guide treatment decisions and improve outcomes^6^, but there exist few standardized protocols for interpreting this complex, information-rich data, leading to variability in how this data is used to recommend treatment^7^.

Previous work has identified features from gait analysis that can predict if a patient will have a positive outcome following an orthopedic surgery. For example, several teams have built models to predict outcomes following psoas lengthenings^8^, rectus femoris transfers^9^, femoral derotation osteotomies^10^, and single-event multilevel surgeries (SEMLS) to improve crouch gait^5^. For the interventions studied, these tools can predict positive outcomes with over 70% accuracy, and consistent applications of the tools would likely lead to improvement on historical positive outcome rates.

These separate models tended to have common outcome predictors, suggesting there are intrinsic patient factors that increase likelihood of positive outcomes, regardless of the specific intervention. Studies predicting the outcome of SEMLS have found that the pre-operative level of the chosen outcome variable is the strongest predictor of post-operative improvement^11,12^. When controlling for this effect, the next strongest predictors were Gross Motor Function Classification Scale (GMFCS)^11^ and dynamic motor control^12^.

Previous studies have provided valuable tools to aid clinicians in formulating treatment plans, but two important issues remain unresolved. First, it is unclear how the expected gains from surgery compare to the expected evolution of gait without surgery. Gough *et al*.^13^ found that patients who did not receive a SEMLS showed a decline in gait function, while patients who had a SEMLS showed improvement, suggesting that the effects of SEMLS may be underestimated. However, there are conflicting findings about how patients’ functional ability and gait evolve without treatment, with some studies reporting improvement and/or maintenance of function with age^14,15^ and others reporting decline in function over time^16,17^. There is agreement that the gait of patients from different functional groups evolve differently over time^14,16,17^.

Second, large, randomized, prospective clinical trials are difficult to conduct due to established standard-of-care practices, as well as the number of permutations of surgeries possible in a SEMLS. As a result, most studies are based on retrospective data in which the patients that receive a SEMLS and those who receive no surgical intervention are often unmatched, and are not representative subsamples of the whole CP population. It is unclear if and how conclusions drawn about effects of surgery and natural progression of gait are biased to the subgroups analyzed. Patients who are at or below their age-matched peers in gait quality or functional ability are much more likely to receive surgical treatment to improve gait. Thus, data used to make predictions on natural progression without treatment or on surgical outcomes are biased to represent patients with higher or lower gait function, respectively. In a systematic review of studies evaluating the effectiveness of SEMLS in treating patients with CP, McGinley *et al*.^18^ found that few studies, if any, were true randomized clinical trials. Thomason *et al*.^19^ performed a pilot randomized study to examine the effects of a SEMLS, but the study only contained 19 patients, which is likely to be too small a sample given the heterogeneity of patient characteristics in the population.

To address these limitations of past research and aid clinical decision-making, we aimed to (i) build and share regression models to predict the effect size of a SEMLS for a limb, quantified as improvement in the Gait Deviation Index (GDI)^20^ at a follow-up visit when controlling for expected progression without surgery, and (ii) use these models to infer patient characteristics that are associated with improved outcomes. We, like other studies, analyzed a retrospective, observational clinical dataset, but used previously reported methods to correct for the sampling bias often seen in such datasets^21,22^. We believe these tools, available as a supplementary download, can increase the rate of good surgical outcomes by aiding in selection of surgical candidates and suggesting pre-operative training interventions that better prime patients for positive outcomes of surgery.

## Methods

### Data

**Figure 1:**
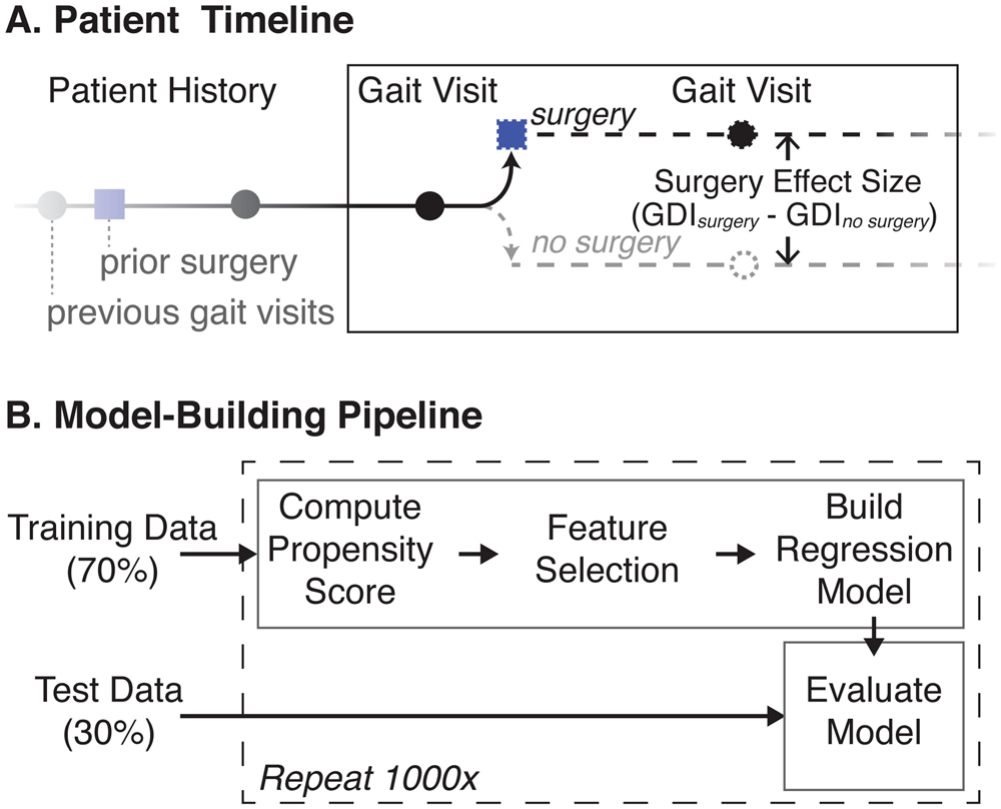
Estimating effect size of surgery. Patients receive care over their lifetime, including gait analyses (●) and surgical interventions (■), to treat abnormal gait. (**A**) The true effect size of surgery is the difference in patient states with and without surgical intervention. Since, after a given gait visit, the patient can continue on only one of the “surgery” or “no surgery” paths, this effect size is unobservable and must be estimated using models built from patient history and gait visit data. (**B**) Data for each patient limb analyzed were first split into training and testing sets. The training data were used to estimate patient propensity for surgery at this center, select features, and build regression models to estimate outcome with and without surgical intervention. The held-out test data were used to evaluate the resultant regression models. Using the fixed training and testing data, these model-building and evaluation methods were repeated 1,000-fold to ensure results were robust to any stochastic variation in the training process.

We retrospectively analyzed the affected limb(s) of ambulatory patients with a diagnosis of CP seen at Gillette Children’s Specialty Healthcare Center for Gait and Motion Analysis. Institutional Review Boards (IRB) at Stanford University and Gillette Children’s Specialty Healthcare both approved this study. Patients, and guardians, where appropriate, gave informed consent at the clinical visit for their data to be included in future studies. In accordance with IRB guidelines, all patient data was de-identified prior to any analysis. To be included, the patient had to receive two gait analyses spaced between 9 and 36 months apart, and be between the ages of 5 and 18 at the initial visit. We identified two groups of patients: the *control* group, who received only conservative treatment (e.g., no surgery or botulinum toxin type A injections) between the gait analyses, and the *surgery* group who received an intervening SEMLS (Fig. 1A). For this study, we defined a SEMLS as having at least one orthopedic surgery on the analyzed side and at least two orthopedic surgeries in a single surgical event. Exploratory or hardware removal surgeries did not contribute to this count. Patients who received only botulinum toxin type A injections between gait visits were not included in this analysis. These criteria yielded 909 limbs in the *control* group and 1,424 limbs in the *surgery* group. Limbs without a full gait analysis at the second gait visit were included in the propensity score model to estimate and correct for bias in treatment assignment (described below), but excluded from the regression models to predict follow-up GDI. This left 582 *control* limbs and 1,133 *surgery* limbs for the regression models.

Candidate features for the regression models to predict second-visit GDI of limbs in each group were derived from the patient history, demographics, initial gait visit kinematic, kinetic, non-dimensional spatio-temporal^23,24^, and physical exam data, and the impending surgical procedures. Joint-level measurements for manual muscle strength, selective motor control, and spasticity from the physical exam data tend to be noisy but correlated^3^; we condensed these into three respective summary scores using an algorithm similar to principal component analysis that allowed for robust imputation of missing measurements^25^. We used the kinematic data to compute muscle-tendon lengths and velocities of six major lower extremity muscles over the gait cycle using musculoskeletal modeling software, OpenSim^26^, and a generic musculoskeletal model^27^. From these, we extracted peak lengths and velocities over the gait cycle as candidate features. Finally, we used singular value decomposition to project the time series data (kinematics, kinetics, and computed muscle-tendon lengths and velocities) into a lower-dimensional space to compute the following summary features: GDI^20^ and GDI-Kinetic^28^, and similar measures based on the muscle-tendon lengths and velocities. The coordinates of these low-dimensional projections were also used as features. This dataset contained few missing values, and any missing values were filled using mean imputation^29^. A full list of candidate features is available in Supplemental Table S1.

### Model Development

To build the models, the data was split into training (70%) and testing (30%) data. With the training data, we first computed the propensity for surgery based on first-visit gait analysis data. These propensity scores were then used to compute weighted regression models to predict second-visit GDI for the *control* and *surgery* groups, based on first-visit gait analysis data. The models were evaluated on the testing data. This pipeline was repeated 1,000-fold to ensure the results were not sensitive to any stochastic processes in the training phase (Fig. 1B). These steps are described in more detail below, and equations to implement the methods are given in the Appendix.

#### Propensity Score

The propensity score^21,22^ is defined as the likelihood of being assigned treatment (in our case, a SEMLS) given the observed characteristics of the subject (in our case, patient history and initial gait visit data). Weighting samples for regression in an observational dataset by the inverse of the propensity score has been proposed as a method to retrospectively account for sampling bias. This method has been used, for example, to examine the effects of physical activity on health^30^ and the effect of statin prescriptions on mortality risk in patients recovering from an acute myocardial infarction^31^.

We estimated the propensity score using a random forest classifier that predicted whether the analyzed limb would go on to receive a SEMLS based on the following characteristics that we hypothesized may bias treatment assignment: patient demographics (age, body-mass index), CP sub-type diagnosis, surgical history (selective dorsal rhizotomy and/or orthopedic), gait severity (ipsilateral and contralateral GDI, normalized walking speed, percent of gait cycle spent in single and double support stance phase), strength, selective motor control, and spasticity (Equation A1). The random forest was trained using the randomForest package in R^32^; we used 100 trees and specified class priors to correct for class imbalance.

#### Regression Model

For each of the *surgery* and *control* regression models, we used *l*_1_-regularization on the standardized regression coefficients to select a sparse subset of features from the candidate feature set (Equation A2). We weighted data using the propensity scores and did a 10-fold cross validation with the training data to choose the optimal regularization weight that resulted in the sparsest model while still maintaining good model performance (Equation A2, notes).

After using regularization to select a sparse set of features, we trained weighted linear models using only these features to estimate the regression coefficients that minimized total weighted squared error between the true and predicted values of second-visit GDI (Equation A3) and estimate regression coefficient covariance (Equation A4).

To estimate the importance of a given variable to outcome (second-visit GDI), we computed standardized effect sizes from the regression coefficients for each variable included in our model. This measure gives the change in predicted GDI per one standard deviation change in the feature variable in the training data. Standardized variances of the effect sizes were similarly computed from the coefficient covariance matrix.

#### Model Testing

On each of the 1,000 model-building iterations, we evaluated the model on the testing data. We used the model to predict second-visit GDI (Equation A5) and computed variance explained (*R*^2^). We also performed a post-hoc classification to see how accurately we could predict which patients would have a follow-up GDI of at least 80, indicating a gait score within 2 standard deviations of healthy gait, and computed the area under the Receiver Operating Characteristic (ROC) curve—the plot of true positive classification rate against the false positive rate as this GDI-threshold is varied—describing the sensitivity-specificity trade-off of varying this GDI-threshold.

#### Estimation of surgery effect size

The *surgery* and *control* regression models give estimates of future GDI with and without surgical intervention. Using these, we computed for all observations the predicted “true” effect size of surgery, defined as the difference in predicted GDIs with and without surgery (Fig. 1A). We also computed the 95% confidence interval of outcome with and without surgery for selected case studies (Equation A5).

## Results

**Figure 2:**
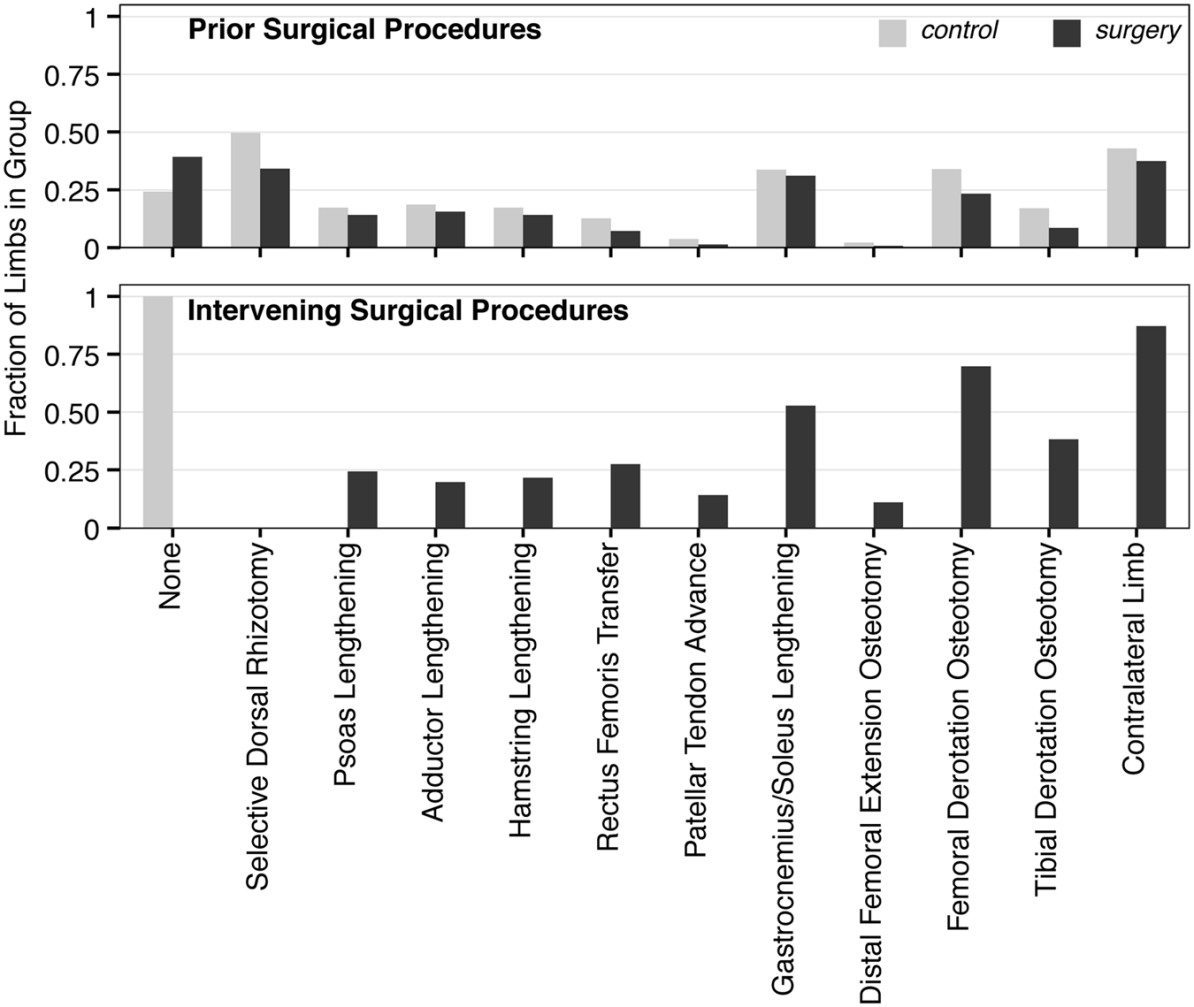
Patient prior and intervening surgical procedures. Both the conservative (*control*) and single-event multi-level surgery (*surgery*) patient cohorts had similar surgical histories prior to the first analyzed gait visit (top). Intervening surgical procedures in the *surgery* patient cohorts were variable, and all analyzed limbs had at least two of the tabulated surgeries. Other specific surgical procedures not tabulated here may have been concurrently performed, but were much less frequent.

On the population level, limbs in the *control* and *surgery* groups both had similar orthopedic surgical histories before the first gait visit analyzed (Fig. 2). Limbs in the *surgery* group had a wide variety of intervening surgical procedures between analyzed gait visits, with 87% of patients having surgery on both limbs (Fig. 2).

**Figure 3:**
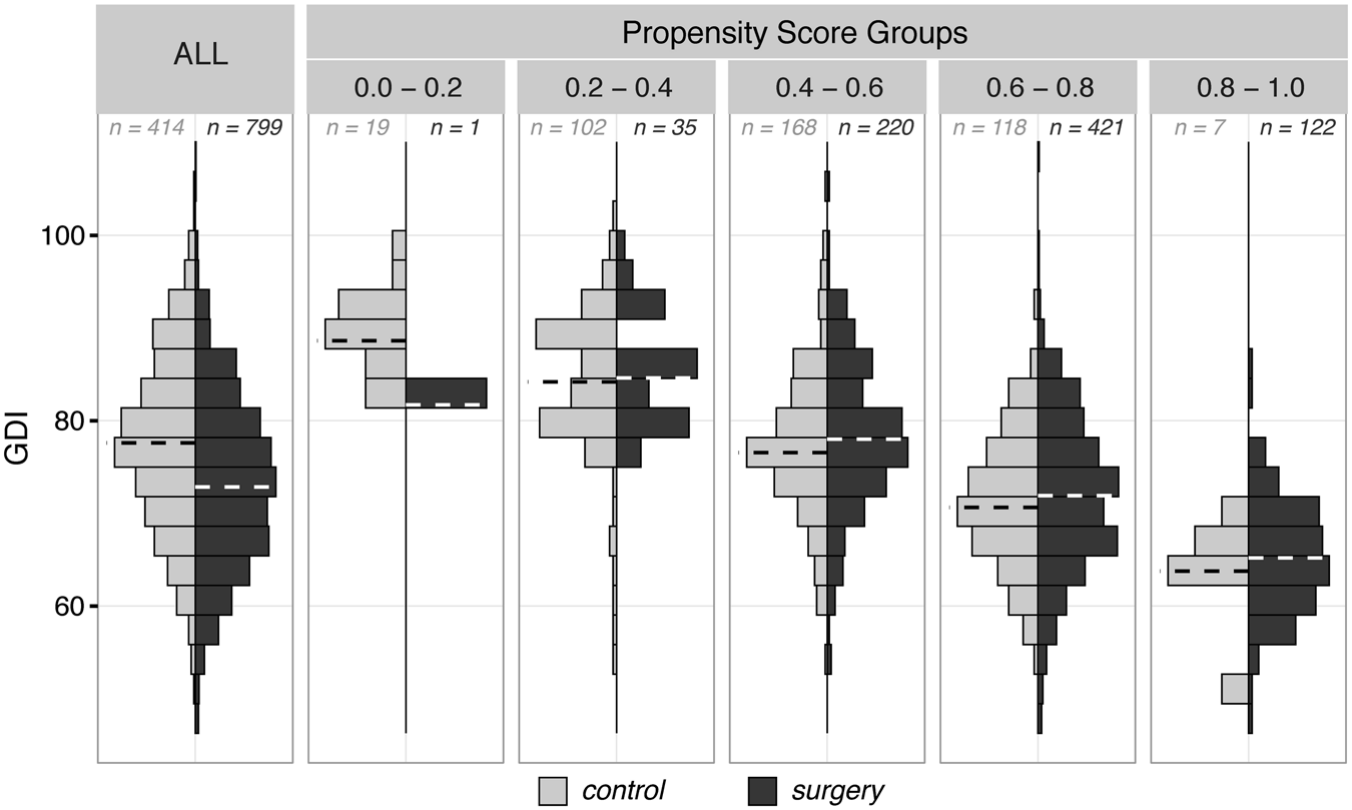
Propensity-score stratified subgroups. Pre-operative gait features, e.g., Gait Deviation Index (GDI) in the *control* (light gray) and *surgery* (dark gray) groups were systematically different, with limbs in the *surgery* group more severely affected, on average, than limbs in the *control* group (ALL panel). However, *control* and *surgery* limbs with similar propensity scores showed similar pre-operative features (‘Propensity Score Groups’ panels). Numbers in each panel indicate number of limbs displayed in each histogram, dashed lines indicate group medians, and histogram heights are normalized to count in each group.

Our propensity score models to predict the likelihood of a limb receiving a SEMLS conditioned on the first-visit gait analysis data were able to, on average, classify limbs as *control* or *surgery* limbs with 67% accuracy, 66% sensitivity, and 71% specificity in the training limbs, and 70% accuracy, 66% sensitivity, and 74% specificity in the testing limbs. When stratified by propensity score, *control* and *surgery* limbs in the training data had similar distributions of pre-operative variables; for example, while the mean GDI of all *control* limbs was 4.4 points higher than that of all *surgery* limbs, the mean GDIs between the *control* and *surgery* limbs in the propensity-score-stratified subgroups differed by only 0–2 points. The notable exception is the low-propensity group (propensity score between 0.0–0.2) where there was a low number of *surgery* limbs (Fig. 3).

**Figure 4:**
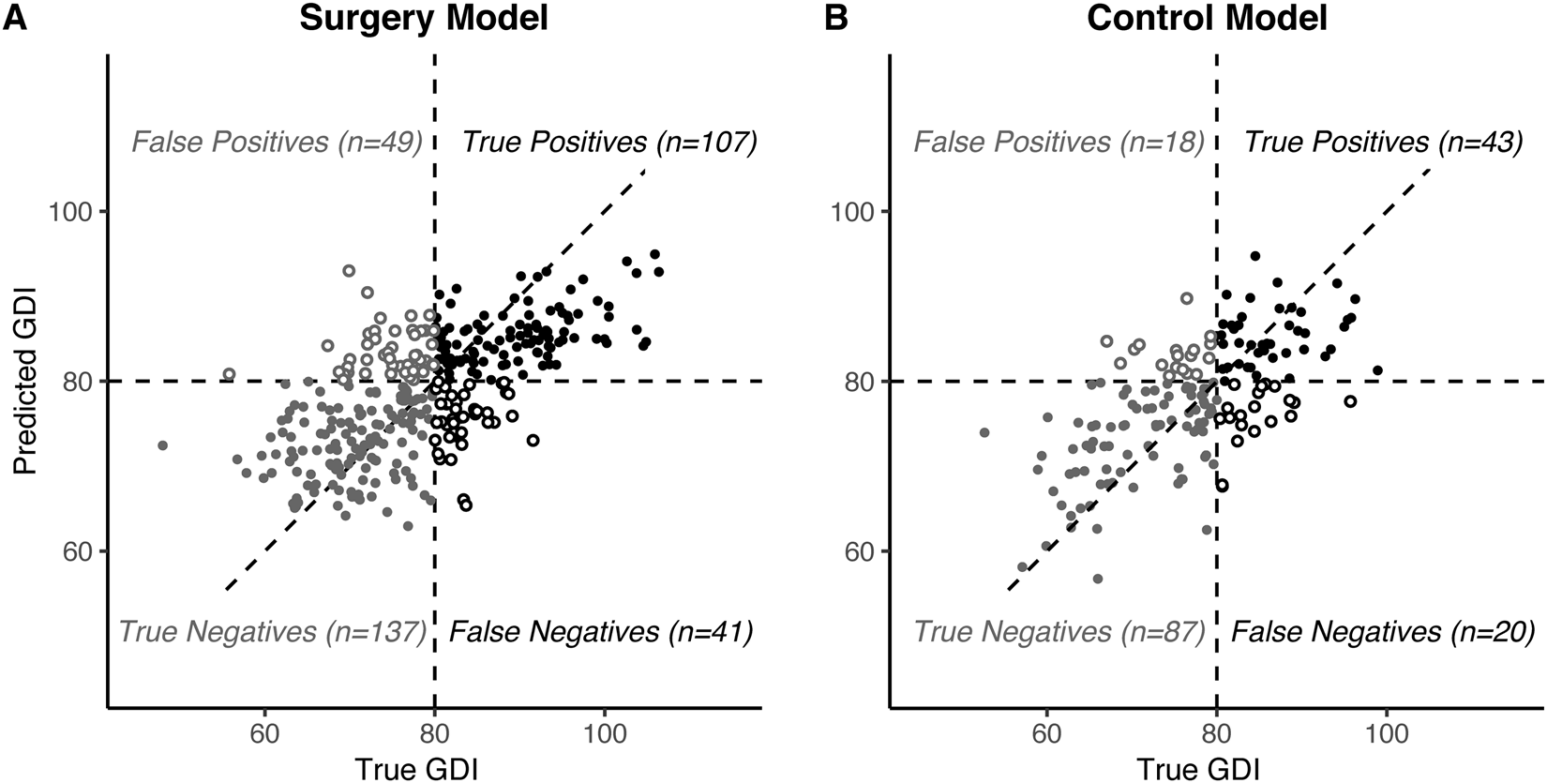
Regression model performance. Linear regression models were built to predict the second-visit GDI using initial gait visit data and intervening treatments for the *surgery* and *control* groups. (**A**) The *surgery* models explained, on average, 41% of variance in outcome. Post-hoc classification of whether post-operative GDI was above 80 had 73% accuracy. The area under the ROC curve was 0.82. (**B**) The *control* models explained, on average, 40% of variance in outcome. Post-hoc classification had 77% accuracy, with area under the ROC curve of 0.81.

The regression models to predict GDI at the follow-up gait analysis visit were able to explain, on average, 46% (train) and 41% (test) of the variance in outcome in the *surgery* limbs, and 59% (train) and 40% (test) of the variance in outcome in the *control* limbs. A post-hoc classification of which limbs had a second-visit GDI of at least 80 had 76% (train) and 73% (test) accuracy in the *surgery* limbs (Fig. 4), and 80% (train) and 77% (test) accuracy in the *control* limbs (Fig. 4). The area under the ROC curve for this post-hoc classification was 0.84 (train) and 0.82 (test) in the *surgery* limbs, and 0.87 (train) and 0.81 (test) in the *control* limbs.

The models to predict second-visit GDI in the *surgery* group included a median of ten variables, with the following eight variables included in at least 80% of models: (1) first-visit GDI, (2) normalized walking speed, (3) selective motor control score, (4) manual muscle strength score, (5) peak ankle plantarflexion moment, (6) contralateral limb GDI, (7) knee flexion angle at initial contact, and (8) diagnosis of quadriplegia. The models to predict second-visit GDI in the *control* group included a median of 5 variables, with the following five variables included in at least 80% of models: (1) first-visit GDI, (2) selective motor control score, (3) contralateral limb GDI, (4) normalized step length, and (5) normalized walking speed. Standardized effect sizes and standard deviations for each variable are given in Table 1.

**Table 1:** Regression model coefficients and effect sizes.

	Variable	Coefficient (SD)	Standardized Effect Size (SD)
*Surgery*	GDI	0.27 (0.032)	2.58 (0.30)
	Normalized Walking Speed	11.50 (2.67)	1.34 (0.31)
	Selective Motor Control Score	4.21 (1.34)	1.15 (0.37)
	Strength Score	5.51 (1.71)	1.12 (0.35)
	Peak Ankle Plantarflexion Moment	1.58 (0.60)	0.73 (0.28)
	Contralateral Limb GDI	0.050 (0.031)	0.51 (0.31)
	Knee Flexion Angle at Initial Contact	−0.059 (0.020)	−0.82 (0.27)
	Diagnosis – Quadriplegia	−2.01 (0.62)	−0.84 (0.26)
	Constant	48.03 (2.30)	—
*Control*	GDI	0.57 (0.030)	5.40 (0.25)
	Selective Motor Control Score	3.71 (0.95)	0.98 (0.25)
	Normalized Step Length	5.67 (1.28)	0.90 (0.20)
	Contralateral Limb GDI	0.096 (0.028)	0.87 (0.25)
	Normalized Walking Speed	4.17 (1.90)	0.50 (0.23)
	Constant	19.99 (2.68)	—

**Figure 5:**
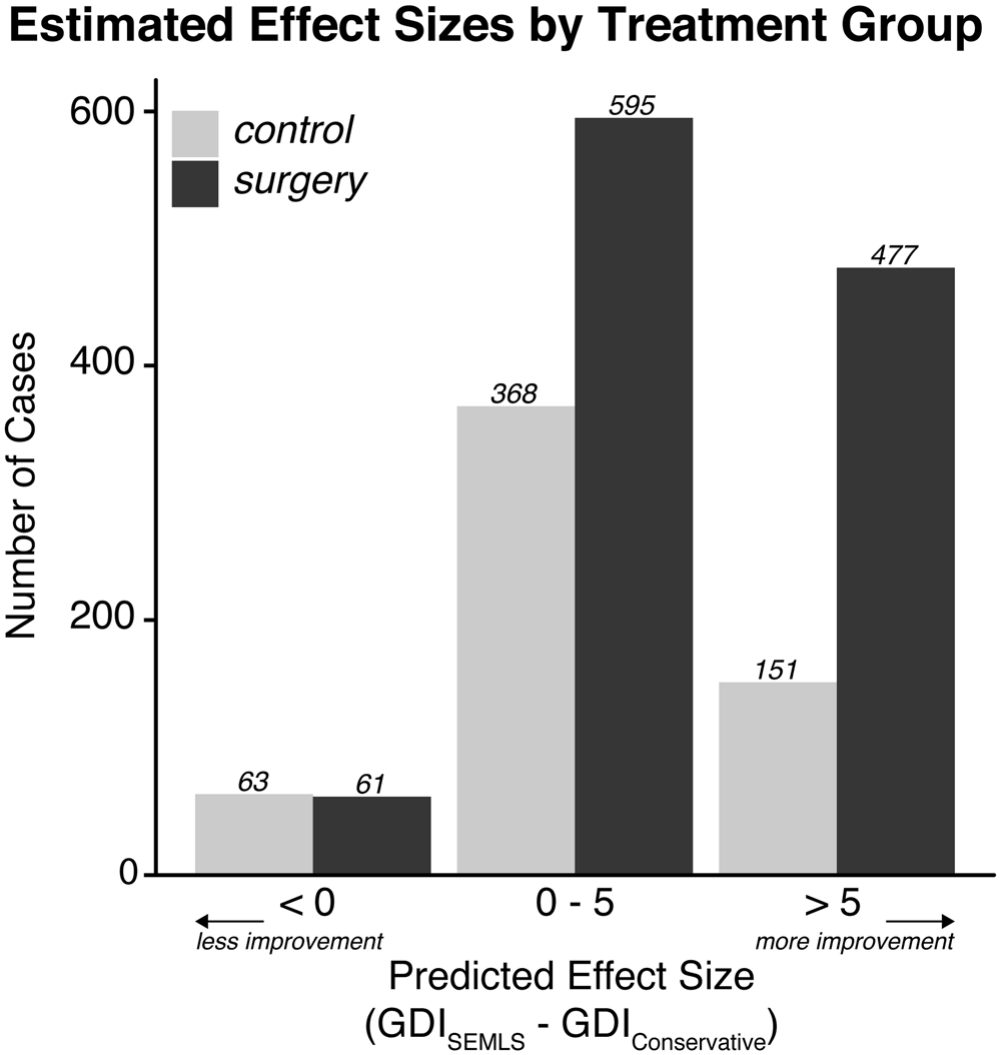
Estimated effect size. We estimated effect size as the difference in expected GDI with and without a SEMLS intervention. A positive effect size indicates improvement following SEMLS. Using the developed regression models, 93% of all limbs (computed from displayed counts) show a positive expected effect size, and 37% show an expected effect size of at least 5.

With these models, we predicted 93% of limbs would have a positive (>0-point improvement in GDI) effect from surgery compared to receiving no intervention, but only 37% would have at least a clinically meaningful 5-point improvement in GDI with surgery compared to no intervention (Fig. 5). Of these limbs that were predicted to have at least a 5-point improvement in GDI with surgery compared to no intervention, 76% received surgery.

## Discussion

**Figure 6:**
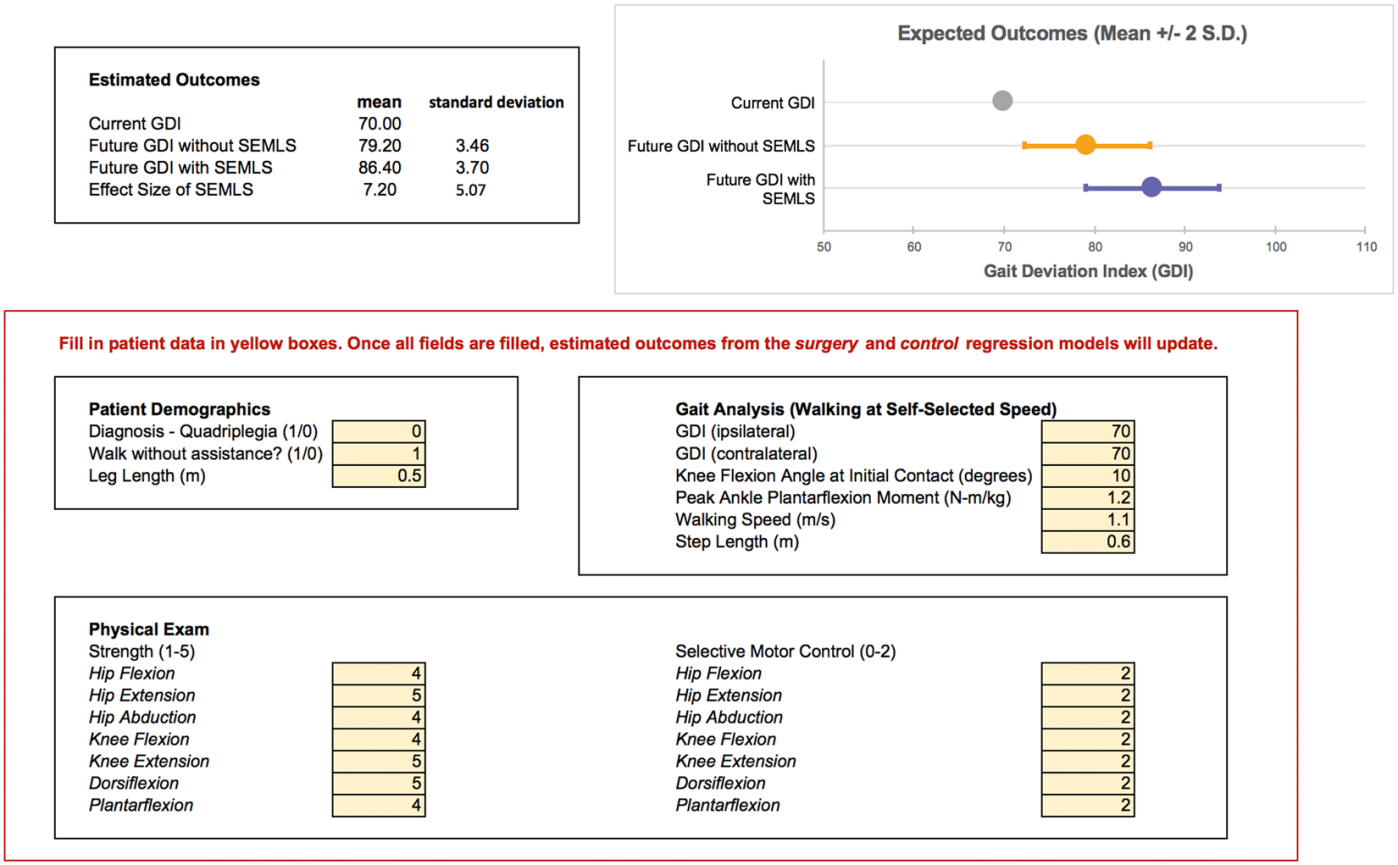
Interactive clinical tool to estimate surgery effect size. The regression models developed in this study are implemented in Microsoft Excel™ and are available to download with the study. The screenshot is used with permission from Microsoft.

In this study, we built regression models to estimate the expected effect of a SEMLS intervention compared to no surgical intervention in ambulatory patients with cerebral palsy. Our models were able to identify with 73% and 77% accuracy which limbs would have a GDI within two standard deviations of typical gait in surgically and non-surgically treated limbs, respectively. We also identified 26% of the *control* (non-surgically treated) population that may have benefited from a SEMLS intervention, and 5% of the *surgery* population that were “over-treated” and were not expected to show any improvement with surgery relative to natural progression without surgery (Fig. 5). We can infer from the selected features in each model the intrinsic patient factors that are important predictors of gait progression with or without surgical intervention, and estimate the role of these factors in determining the true effect size of SEMLS. Finally, these features identify potential points of conservative intervention with physical therapy before surgery to improve upcoming surgical outcomes. These models are available to the clinical community to use as an evaluation tool when considering new surgical candidates (Supplemental worksheet and Fig. 6).

We define the “true” effect size of surgery as the difference between the patient’s GDI, with and without surgery (Fig. 1A). This is, by nature, unobservable, but can be estimated by subtracting the expected GDI without surgery, obtained from the *control* model, from the expected GDI with surgery, obtained from the *surgery* model. Thus, patient characteristics (regression model features) with either a positive effect on GDI with surgery or a negative effect on GDI without surgery will increase the effect size of surgery, and vice versa. For features that are shared in both the *surgery* and *control* models, the difference in the variable coefficients will determine whether the feature has a positive or negative influence on the surgery effect size.

In both models, lower gait severity, measured by GDI, and greater selective motor control assessed in the physical exam at the initial visit had strong, positive effects on the follow-up visit GDI (Table 1). These results are consistent with previous studies that have shown similar results with various measures of outcome, including GDI^11,12^. However, higher first-visit GDI decreases the effect size of surgery. This idea of diminishing returns—that patients with higher gait function and less room to improve will tend to show lesser gains from surgery—has been consistently shown in other studies as well^5,11,12,33^. Increased selective motor control, on the other hand, increases the effect size of surgery.

When controlling for first-visit GDI and selective motor control, in the *surgery* model, normalized walking speed had the strongest effect on second-visit GDI. In our model, walking speed may be a surrogate measure for level of neurologic involvement^34^ and postural balance^35^, with higher walking speeds associated with improved function. Faster walking speeds also had a positive effect on second-visit GDI in the *control* model, but the effect was not as large as expected after surgery.

Finally, greater strength and higher peak ankle plantarflexion moment increased the effect size of surgery. These variables may be a measure of ability to support and propel oneself during the stance phase of gait, with stronger patients tending to show better recovery from surgery.

The remaining predictor variables decreased the effect size of surgery, either because they had negative effects on follow-up GDI with surgery, or because they had positive effects on follow-up GDI without surgery. Greater contralateral limb GDI increased follow-up GDI with or without surgery, but resulted in a net decrease in surgery effect size. Increased crouch (greater knee flexion angle at initial contact) and a diagnosis of quadriplegia each had a negative effect on GDI after surgery but did not affect gait progression without surgery. Longer normalized step lengths, on the other hand, had a positive effect on follow-up GDI without surgery, and hence diminished the relative gains from surgery. Similar to normalized walking speed, step length may be an indicator of postural balance and stability in single limb stance which are important in gait^1^.

Encouragingly, some of the discussed parameters, such as postural balance and strength, can be improved with training^36^ and physical therapy^37^. Future controlled studies are warranted to test if targeted therapy to improve these parameters can help both improve outcome without surgery and increase the effect of surgery relative to natural progression.

**Figure 7:**
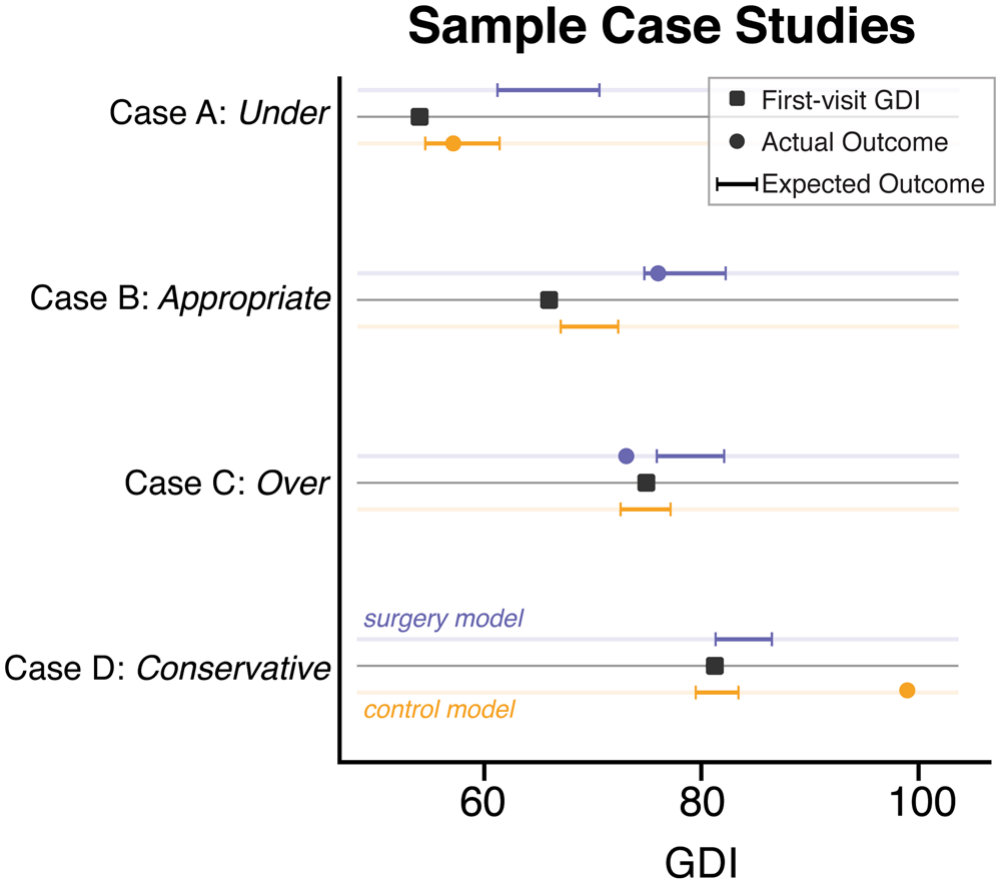
Case studies. Four examples of varying first-visit severity were selected, illustrating cases where our models suggest limbs were (**A**) under-treated, (**B**) appropriately surgically treated, (**C**) potentially over-treated, and (**D**) potentially conservatively treated. Horizontal colored bands represent 95% confidence interval for range of expected outcomes without (orange) and with (blue) surgery. Black squares represent GDI at first gait visit, and colored dots represent GDI at follow-up gait visit.

The *surgery* and *control* models together estimate the effect of surgical intervention when controlling for expected changes due to natural progression without surgery. Figure 7 illustrates how these models applied to four case studies from our dataset could have been used to guide treatment decisions. Cases A and B both were expected to improve without surgical intervention (*control* model), but show even more improvement with a SEMLS (*surgery* model). The estimated true effect size of surgery for both cases may be large enough to warrant surgical intervention. While Case B went on to receive a SEMLS after the initial gait visit, Case A did not. The observed outcomes suggest that Case A was under-treated, while case B was appropriately treated. Cases C and D were not expected to show much change without surgical intervention (*control* model), and show only small improvements with a SEMLS (*surgery* model). These results suggest the true effect size of surgery may be too small to warrant surgical intervention. Case C went on to receive a SEMLS, but did not show improvement in GDI at the follow-up visit and may have been over-treated. Case D was conservatively treated and did not receive surgical intervention, and showed greater improvements than our *control* model predicted.

There are many factors, including differences in clinical philosophy (aggressive intervention vs. wait-and-watch) and family preferences that can drive treatment decisions, and future studies may be warranted to further understand what influences treatment decisions in addition to predicted outcomes. In this study, we only analyzed data from a single clinical center. While we do not expect that our results are strongly biased as a result of the center’s treatment philosophies, it would be valuable for other centers to retrain this model and share conclusions learned from their own data. Nevertheless, given our estimate that only 37% of limbs will show a meaningful improvement with SEMLS compared to natural progression, we believe the regression models presented in this study can, with further development and validation across multiple treatment centers, be used to identify good surgical candidates and avoid over- or under-treatment.

There are several limitations and extensions of our work that should be considered before clinical implementation. First, while our *surgery* and *control* models explain 41% and 40% of variance in outcome, respectively, and suggest patient characteristics important for general surgical recovery and gait progression, there is still a significant portion of variance in outcome that is left unexplained. Some of this unexplained variance is likely associated with differences in how patients respond to specific surgical procedures. For example, when looking at only the subset of limbs that underwent a hamstrings lengthening surgery, including peak hamstrings length and velocity as a predictive variable in the *surgery* model may increase the amount of variance explained^38^. We expect that future work focusing on a cohort of patients who received a specific surgery and conditioning on the results presented in our study will be able to (i) explain some of the remaining variance and (ii) identify procedure-specific indicators for optimizing outcomes. These studies, along with past work, can provide standardized clinical protocols to recommend treatments, set patient expectations, and improve surgical outcomes.

In addition to procedure-specific predictors of outcomes, other social and environmental factors can strongly affect patient response to surgery. In our study, we analyzed the predictive capacity of quantitative, biomechanical features of gait on the effect size of surgery. We did not analyze, however, other qualitative and equally important factors such as (i) quality of inpatient and outpatient post-operative rehabilitation, including availability and access to physical therapy, (ii) patient motivation, and (iii) family and school support. Various protocols have been suggested as part of conservative treatment and as part of post-operative rehabilitation to maximize recovery of strength and function following orthopedic surgery, and should be integrated when formulating a treatment plan^1,39,40^.

Second, sample reweighting is a helpful technique to correct for sampling bias in a retrospective, observational dataset such as ours where the data is predictably biased due to standard-of-care treatment decisions. Despite these corrections, constructing robust, unbiased predictive models of both surgical outcomes and natural progression remains a challenge. In our study, we reweighted samples by the inverse of their propensity of receiving their actual treatment decision (i.e., *surgery* limbs that were unlikely to receive surgery were upweighted in the *surgery* regression model, and vice versa for the *control* limbs). Even with this correction, biases remain. For example, our estimates of how severely affected patients will progress without surgical intervention were still based on a relatively small sample of data. Furthermore, there are likely to be other variables not included in our propensity score balancing that affect outcome (e.g., quality of physical therapy) and likely differ between the two groups studied.

If the analyses presented in this study were redone without the sample reweighting, we found similar performance in the regression models (41% and 37% variance explained in the *surgery* and *control* models, respectively), but with more selected features in the *l*_1_-regularization phase. It is unclear if these extra selected features were a consequence of the slight biases in the training data that sample reweighting methods aim to correct. Nevertheless, the additional features had a smaller effect than those included in the weighted models. It is encouraging that, even with our more conservative modeling approach in rebalancing the dataset, we were able to match performance from models built using standard practices and with a sparser set of final features.

Finally, our study does not address short-term gains in functional metrics other than the GDI, nor long-term implications for gait and function into adulthood. While the GDI is a well-validated objective and reproducible metric that has been shown to have good association with functional assessment scores^20^, it is not a direct measurement of functional capacity. Furthermore, factors such as accelerated joint deterioration and joint pain that may result from chronic abnormal walking patterns^41^ may result in patients becoming less ambulatory later in life^42,43^ even with positive orthopedic treatment in childhood. The elapsed time between the initial and follow-up gait visits was not selected as a predictor of GDI at the follow-up visit in neither our *surgery* nor *control* regression models, which suggests that this loss of mobility is not a significant factor in our estimates of short-term surgery effect size. However, longitudinal studies that have examined patient outcomes after, e.g., single-event multilevel^44^, gastrocnemius lengthening^45^, and distal femoral extension osteotomy and patellar tendon advance^46^ surgeries have reported some, but not all, benefits of surgery are maintained long term. While our model provides a tool to assess potential short-term (1–3 years) gains in mobility, it is only one piece of the clinical decision-making paradigm^47^. Both short-term and long-term mobility and functional goals of the patient and family should be considered while making a treatment plan.

## Conclusion

We have built and shared regression models to predict the effect of surgery in improving gait in patients with cerebral palsy when controlling for expected natural progression. These models are available to download with the study, and can help identify patients who are good surgical candidates and ultimately improve the rate of positive outcomes. The methods used here can be used to build other predictive models focused on estimating effects from specific surgical procedures, though we suggest conditioning predicted outcomes on the effect of the intrinsic patient factors identified here. Finally, we suspect features selected in our models are not center-specific, but it is unknown how well our models extend to different treatment centers. We encourage other centers to, when possible, retrain the models using their own patient data.

## Electronic supplementary material

Surgery Effect Size Worksheet

Supplementary Information

## Data Availability

The trained regression models to predict follow-up GDI for the *control* and *surgery* groups are available with supplemental materials. The dataset analyzed in this study was shared by Gillette Specialty Healthcare under a data-sharing agreement between Stanford University and Gillette Specialty Healthcare. These data are not publicly available due to restrictions on sharing patient health information but are available from the authors on reasonable request and with permission of Gillette Specialty Healthcare.

## References

[1] Gage, J. R., Schwartz, M. H., Koop, S. E. & Novacheck, T. F. *The Identification And Treatment Of Gait Problems In Cerebral Palsy*. John Wiley & Sons (2009).

[2] Wren Tishya A. L., Rethlefsen Susan, Kay Robert M. (2005). Prevalence of Specific Gait Abnormalities in Children With Cerebral Palsy. Journal of Pediatric Orthopaedics.

[3] Rozumalski A, Schwartz MH (2009). Crouch gait patterns defined using k-means cluster analysis are related to underlying clinical pathology. Gait Posture.

[4] Rethlefsen SA, Blumstein G, Kay RM, Dorey F, Wren TAL (2017). Prevalence of specific gait abnormalities in children with cerebral palsy revisited: influence of age, prior surgery, and Gross Motor Function Classification System level. Dev. Med. Child Neurol..

[5] Hicks JL, Delp SL, Schwartz MH (2011). Can biomechanical variables predict improvement in crouch gait?. Gait Posture.

[6] DeLuca PA, Davis RB, Ounpuu S, Rose S, Sirkin R (1997). Alterations in surgical decision making in patients with cerebral palsy based on three-dimensional gait analysis. J. Pediatr. Orthop..

[7] Skaggs DL (2000). Variability in gait analysis interpretation. J. Pediatr. Orthop..

[8] Schwartz MH, Rozumalski A, Truong W, Novacheck TF (2013). Predicting the outcome of intramuscular psoas lengthening in children with cerebral palsy using preoperative gait data and the random forest algorithm. Gait Posture.

[9] Reinbolt JA, Fox MD, Schwartz MH, Delp SL (2009). Predicting outcomes of rectus femoris transfer surgery. Gait Posture.

[10] Schwartz MH, Rozumalski A, Novacheck TF (2014). Femoral derotational osteotomy: surgical indications and outcomes in children with cerebral palsy. Gait Posture.

[11] Rutz E, Donath S, Tirosh O, Graham HK, Baker R (2013). Explaining the variability improvements in gait quality as a result of single event multi-level surgery in cerebral palsy. Gait Posture.

[12] Schwartz MH, Rozumalski A, Steele KM (2016). Dynamic motor control is associated with treatment outcomes for children with cerebral palsy. Dev. Med. Child Neurol..

[13] Gough M, Eve LC, Robinson RO, Shortland AP (2004). Short-term outcome of multilevel surgical intervention in spastic diplegic cerebral palsy compared with the natural history. Dev. Med. Child Neurol..

[14] Rosenbaum PL (2002). Prognosis for gross motor function in cerebral palsy: Creation of motor development curves. J. Am. Med. Assoc..

[15] Rose GE, Lightbody KA, Ferguson RG, Walsh JC, Robb JE (2010). Natural history of flexed knee gait in diplegic cerebral palsy evaluated by gait analysis in children who have not had surgery. Gait Posture.

[16] Bell Katharine J., Õunpuu Sylvia, DeLuca Peter A., Romness Mark J. (2002). Natural Progression of Gait in Children With Cerebral Palsy. Journal of Pediatric Orthopaedics.

[17] Hanna S, Rosenbaum P (2009). Stability and decline in gross motor function among children and youth with cerebral palsy aged 2 to 21 years. Dev. Med. Child Neurol..

[18] McGinley JL (2012). Single-event multilevel surgery for children with cerebral palsy: A systematic review. Dev. Med. Child Neurol..

[19] Thomason P (2011). Single-Event Multilevel Surgery in children with spastic diplegia. J. Bone Jt. Surgery-American Vol..

[20] Schwartz MH, Rozumalski A (2008). The Gait Deviation Index: a new comprehensive index of gait pathology. Gait Posture.

[21] Rosenbaum PR, Rubin DB (1983). The central role of the propensity score in observational studies for causal effects. Biometrika.

[22] Austin PC (2011). An introduction to propensity score methods for reducing the effects of confounding in observational studies. Multivariate Behav. Res..

[23] Hof AL (1996). Scaling gait data to body size. Gait Posture.

[24] Schwartz MH, Rozumalski A, Trost JP (2008). The effect of walking speed on the gait of typically developing children. J. Biomech..

[25] Udell M, Horn C, Zadeh R, Boyd S (2016). Generalized low rank models. Found. Trends® Mach. Learn..

[26] Delp SL (2007). OpenSim: Open source to create and analyze dynamic simulations of movement. IEEE Trans. Biomed. Eng..

[27] Rajagopal Apoorva, Dembia Christopher L., DeMers Matthew S., Delp Denny D., Hicks Jennifer L., Delp Scott L. (2016). Full-Body Musculoskeletal Model for Muscle-Driven Simulation of Human Gait. IEEE Transactions on Biomedical Engineering.

[28] Rozumalski A, Schwartz MH (2011). The GDI-Kinetic: A new index for quantifying kinetic deviations from normal gait. Gait Posture.

[29] Hastie, T., Tibshirani, R. & Friedman, J. *The elements of statistical learning: data mining, inference, and prediction, 2*^nd^*ed.* (Springer Series in Statistics, 2009).

[30] Althoff, T. *et al*. Dose response relationships between physical activity and health using propensity scores. In *Neural Information Processing Systems Workshop on Machine Learning for Health (NIPS ML4H)* (2016).

[31] Austin PC (2014). The use of propensity score methods with survival or time-to-event outcomes: reporting measures of effect similar to those used in randomized experiments. Stat. Med..

[32] Breiman L (2001). Random Forests. Mach. Learn..

[33] Dreher T, Wolf S, Braatz F, Patikas D, Döderlein L (2007). Internal rotation gait in spastic diplegia–critical considerations for the femoral derotation osteotomy. Gait Posture.

[34] Damiano DL, Abel MF (1996). Relation of gait analysis to gross motor function in cerebral palsy. Dev. Med. Child Neurol..

[35] Abd El-Kafy EM, El-Basatiny HMYM (2014). Effect of postural balance training on gait parameters in children with cerebral palsy. Am. J. Phys. Med. Rehabil..

[36] Shumway-Cook A, Hutchinson S, Kartin D, Price R, Woollacott M (2003). Effect of balance training on recovery of stability in children with cerebral palsy. Dev. Med. Child Neurol..

[37] Dodd KJ, Taylor NF, Graham HK (2003). A randomized clinical trial of strength training in young people with cerebral palsy. Dev. Med. Child Neurol..

[38] Arnold AS, Liu MQ, Schwartz MH, Ounpuu S, Delp SL (2006). The role of estimating muscle-tendon lengths and velocities of the hamstrings in the evaluation and treatment of crouch gait. Gait Posture.

[39] Damiano DL (2006). Activity, activity, activity: rethinking our physical therapy approach to cerebral palsy. Phys. Ther..

[40] Seniorou M, Thompson N, Harrington M, Theologis T (2007). Recovery of muscle strength following multi-level orthopaedic surgery in diplegic cerebral palsy. Gait Posture.

[41] Steele KM, Demers MS, Schwartz MH, Delp SL (2012). Compressive tibiofemoral force during crouch gait. Gait Posture.

[42] Murphy KP, Molnar GE, Lankasky K (1995). Medical and functional status of adults with cerebral palsy. Dev. Med. Child Neurol..

[43] Bottos M, Gericke C (2003). Ambulatory capacity in cerebral palsy: prognostic criteria and consequences for intervention. Dev. Med. Child Neurol..

[44] Õunpuu S, Solomito M, Bell K, DeLuca P, Pierz K (2015). Long-term outcomes after multilevel surgery including rectus femoris, hamstring and gastrocnemius procedures in children with cerebral palsy. Gait Posture.

[45] Dreher T (2012). Long-term results after gastrocnemius-soleus intramuscular aponeurotic recession as a part of multilevel surgery in spastic diplegic cerebral palsy. J. Bone Joint Surg. Am..

[46] Boyer ER (2018). Long-term outcomes of distal femoral extension osteotomy and patellar tendon advancement in individuals with cerebral palsy. J. Bone Jt. Surg..

[47] Steiner WA (2002). Use of the icf model as a clinical problem-solving tool in physical therapy and rehabilitation medicine. Phys. Ther..

