# Supplementary material for: Estimating the effect size of surgery to improve walking in children with cerebral palsy from retrospective observational clinical data
Source: Sci Rep. 2018 Nov 5;8:16344. doi: 10.1038/s41598-018-33962-2 (PMC6218552; doi:10.1038/s41598-018-33962-2)
Supplement: Supplementary file 2 — Supplementary Information [file 41598_2018_33962_MOESM2_ESM.pdf]

SUPPLEMENTARY INFORMATION FOR:

Estimating the effect size of surgery to improve walking in children with cerebral palsy from retrospective observational clinical data

Apoorva Rajagopal[1*], Łukasz Kidziński[2], Alec S. McGlaughlin[2], Jennifer L. Hicks[2], Scott L. Delp[1,2], Michael H. Schwartz[3,4]

[1]Department of Mechanical Engineering, Stanford University, Stanford, CA, USA

[2]Department of Bioengineering, Stanford University, Stanford, CA, USA

[3]Center for Gait and Motion Analysis, Gillette Children's Specialty Healthcare, St. Paul, MN, USA

[4]Department of Orthopedic Surgery, University of Minnesota, Minneapolis, MN, USA

* Please direct correspondences to Apoorva Rajagopal (apoorvar@stanford.edu)

APPENDIX Equations to implement propensity and regression models

Propensity score

The propensity score (p) for a limb is the probability of that limb undergoing a specified treatment (T) conditioned on pre-treatment variables (X). For the *surgery* model, the specified treatment assignment was a SEMLS ($i = 1$), and for the *control* model, the treatment assignment was only conservative treatment ($i = 0$):

$$p = P(T = i|X). \quad \text{(A1)}$$

Feature selection

The l_1-regularized model error was defined as:

$$J = \sum_k w_k(y_k - (c_0 + \tilde{c}^T \tilde{x}_k))^2 + \lambda|\tilde{c}|, \quad \text{(A2)}$$

where

$$w_k = \max\left(\frac{1}{p_k}, 20\right)$$

$$y_k = \text{ GDI at follow-up visit for limb } k$$

$$\tilde{x}_k = \text{ vector of 0-mean, 1-variance standardized features variables for limb } k$$

$$\tilde{c}, c_0 = \text{ unknown feature coefficients and constant term, and}$$

$$\lambda = \text{ regularization weight}$$

To select features for the regression models, we chose the largest λ such that the mean 10-fold cross validation error, J, was within 1 standard deviation of the minimum mean cross-validation error. The selected features were those corresponding to the resulting non-zero coefficients, $\tilde{c}$.

Regression model

The regression coefficients for the chosen features, x^*, were computed as

$$c, c_0 = \text{argmin} \sum_k w_k(y_k - (c_0 + c^T x_k^*))^2. \quad \text{(A3)}$$

Covariance of the coefficients were computed as

$$\Sigma_{c,c_0} = \sigma^2\left({X^*}^T W X^*\right)^{-1}, \quad \text{(A4)}$$

where X^* is the matrix containing the n_f selected features for all observations, W is a diagonal matrix of observation weights, and

$$\sigma^2 = \frac{1}{\sum w_k - n_f - 1} \sum_k w_k(y_k - (c_0 + c^T x_k^*))^2.$$

New predictions

For a new observation with features, x, we estimate outcome, y, as

$$y \sim \mathcal{N}\left(c_0 + c^T x,\ x^T \Sigma_{c,c_0} x\right). \quad \text{(A5)}$$

SUPPLEMENTARY TABLE S1 Candidate feature variables.

Data Source (data type)	Variables
Kinematics (continuous)	Pelvic tilt – mean; hip flexion– initial contact; hip flexion– foot-off; hip flexion– mean extension velocity in stance; hip adduction– initial contact; hip rotation– mean; knee flexion– initial contact; knee flexion – mean stance; knee flexion – peak extension; knee flexion – peak flexion; knee flexion – mean extension velocity in swing; ankle dorsiflexion – initial contact; ankle dorsiflexion – peak in stance; ankle dorsiflexion – time of peak in stance (normalized); ankle dorsiflexion – peak in swing; foot progression – mean stance
Kinetics (continuous)	Hip extension moment – peak stance; hip flexion moment – peak stance; hip abduction moment – peak stance; ankle plantarflexion moment – peak; ankle positive power – peak; ankle negative power – peak
Temporal/Spatial (continuous)	Percent gait cycle in stance; percent gait cycle in single stance; walking speed (normalized); step length (normalized); cadence (normalized); step length asymmetry; stance asymmetry
Physical exam (continuous)	Femoral anteversion angle; thigh-foot angle; popliteal angle; knee flexion contracture; ankle dorsiflexion angle – peak with knee extended; ankle dorsiflexion angle – peak with knee flexed 90°; selective motor control score; strength score; spasticity score; selective motor control asymmetry; strength asymmetry; spasticity asymmetry
Patient history (continuous, binary)	Age; elapsed time between initial and follow-up gait visit; body-mass index; diagnosis (triplegic, quadriplegic); delivery weeks premature; walk without assistive device (1/0); had previous surgery (1/0); had previous selective dorsal rhizotomy (1/0); had previous major orthopedic surgery (1/0)
Musculoskeletal model (continuous)	Peak muscle-tendon lengths for lateral gastrocnemius, soleus, psoas, semimembranosus, rectus femoris, vastus medialis; peak muscle-tendon velocities (*z*-scores, normalized to typical gait) for lateral gastrocnemius, soleus, psoas, semimembranosus, rectus femoris, vastus medialis
Low-dimensional representations of time series data (continuous)	Gait Deviation Index (ipsilateral and contralateral), coordinates of kinematics projected into 10-dimensional subspace; Gait Deviation Index – Kinetic; muscle-tendon length deviation index, coordinates of muscle-tendon lengths projected into 1-dimensional subspace; muscle-tendon velocity deviation index, coordinates of muscle-tendon velocities projected into 1-dimensional subspace
Upcoming surgeries (binary)	Ipsilateral or contralateral adductor lengthening, gastrocnemius or soleus lengthening, hamstring lengthening, patellar tendon advance, psoas lengthening, rectus femoris transfer, distal femoral extension osteotomy, femoral derotation osteotomy, tibial derotation osteotomy; selective dorsal rhizotomy
